# Chromatin Liquid–Liquid Phase Separation (LLPS) Is Regulated by Ionic Conditions and Fiber Length

**DOI:** 10.3390/cells11193145

**Published:** 2022-10-06

**Authors:** Qinming Chen, Lei Zhao, Aghil Soman, Anastasia Yu Arkhipova, Jindi Li, Hao Li, Yinglu Chen, Xiangyan Shi, Lars Nordenskiöld

**Affiliations:** 1 School of Biological Sciences, Nanyang Technological University, Singapore 637551, Singapore; qmchen@ntu.edu.sg (Q.C.); asoman@ntu.edu.sg (A.S.); 2 Department of Biology, Shenzhen MSU-BIT University, Shenzhen 518172, China; 6420220005@smbu.edu.cn (L.Z.); anastasia-yu.arkhipova@yandex.ru (A.Y.A.); 2120210065@smbu.edu.cn (J.L.); 2120210064@smbu.edu.cn (H.L.); 2120210061@smbu.edu.cn (Y.C.); 3 Biological Faculty, Lomonosov Moscow State University, 119991 Moscow, Russia

**Keywords:** ionic conditions, chromatin fiber length, confocal fluorescence microscopy

## Abstract

The dynamic regulation of the physical states of chromatin in the cell nucleus is crucial for maintaining cellular homeostasis. Chromatin can exist in solid- or liquid-like forms depending on the surrounding ions, binding proteins, post-translational modifications and many other factors. Several recent studies suggested that chromatin undergoes liquid–liquid phase separation (LLPS) in vitro and also in vivo; yet, controversial conclusions about the nature of chromatin LLPS were also observed from the in vitro studies. These inconsistencies are partially due to deviations in the in vitro buffer conditions that induce the condensation/aggregation of chromatin as well as to differences in chromatin (nucleosome array) constructs used in the studies. In this work, we present a detailed characterization of the effects of K^+^, Mg^2+^ and nucleosome fiber length on the physical state and property of reconstituted nucleosome arrays. LLPS was generally observed for shorter nucleosome arrays (15-197-601, reconstituted from 15 repeats of the Widom 601 DNA with 197 bp nucleosome repeat length) at physiological ion concentrations. In contrast, gel- or solid-like condensates were detected for the considerably longer 62-202-601 and lambda DNA (~48.5 kbp) nucleosome arrays under the same conditions. In addition, we demonstrated that the presence of reduced BSA and acetate buffer is not essential for the chromatin LLPS process. Overall, this study provides a comprehensive understanding of several factors regarding chromatin physical states and sheds light on the mechanism and biological relevance of chromatin phase separation in vivo.

## 1. Introduction

In eukaryotes, our genome comprising approximately two meters of DNA in humans is packed in the confined volume of the cell nucleus in the form of chromatin. The fundamental central unit of chromatin is the nucleosome core particle (NCP), a complex of about 145–147 bp DNA and an octamer of two copies of each of the four histone proteins, H2A, H2B, H3, and H4 [1,2]. The positively charged N-termini of all eight histones protrude out of the core region of the histone octamer (HO) and can mediate nucleosome–nucleosome interactions. The NCPs in the chromatin fiber are separated by linker DNA with differing (about 150–230 bp) nucleosome repeat lengths (NRLs) [3]. Chromatin is a highly dynamic complex at both the mesoscale [4,5,6] and nanoscale [7,8,9], which is well-regulated to maintain cellular homeostasis. Among those dynamic features, solid and liquid phase separation and transition have received much attention, but the underlying mechanism and functional relevance are not fully understood [10,11,12,13].

Interphase chromatin in vivo is organized into different chromosome territories in the nucleus [14] that are occupied by chromosomes. The chromosome region may be considered to consist of independent and dynamic topologically associating domains (TADs) [15]. In human nuclei, TADs are around one-megabase-long chromosomal fragments of several hundred nm in size [16]. Nucleosome positioning experiments via genome-wide deep sequencing [17] and cryo-electron tomography study of mitotic chromosomes [18] suggested that they are mostly heterogeneous and largely lack the regular positioning of nucleosomes. However, quantitative super-resolution nanoscopy revealed that nucleosomes tend to assemble in clutches formed by groups of 4–10 nucleosomes [19].

Based on in vivo work that implied the condensation of chromatin into a 30 nm fiber structure [20,21], the classic model of chromatin structure emerged: NCPs are connected by linker DNA and are compacted (folded) into 30 nm fibers. In recent years, much progress in understanding of this fiber structure was obtained by Cryo-EM studies of in vitro reconstituted nucleosome arrays from the Widom high-affinity 601 nucleosome positioning sequence [22,23], which enables the preparation of arrays of regularly spaced nucleosomes with well-defined NRLs and numbers of nucleosome units. The folded chromatin structures of these nucleosome arrays can be characterized by either a two-start zig-zag or one-start solenoid structure, depending on the NRLs [24]. On the basis of several studies, the presence and significance of this ordered 30 nm fiber were put into question as generally relevant for the in vivo situation [19,25,26]. Alternatively, chromatin condensation has been suggested to proceed into an irregular folding of chromatin fibers. A recent study regarding the chromatin structure during salt-dependent compaction in vitro and in vivo suggested that 30 nm fibers could be an intermediate state between the 10 nm fiber and supra-molecular oligomers with so-called interdigitated 10 nm fibers [27].

Chromatin is a polycation–polyanion complex, where the histones contribute positive charges, and DNA possesses negative charges that are only half neutralized by the histones. Consequently, DNA–DNA repulsion is a key factor in chromatin compaction. The particular roles of cations in mediating NCP–NCP stacking and chromatin fiber folding in vitro have been addressed [28]. Biophysical studies of native and in vitro reconstituted chromatin fibers demonstrated their polyelectrolyte behavior in the presence of multivalent cations that induce chromatin condensation and re-dissolution at high cation concentration [29,30,31], with electrostatic interactions governing the intramolecular folding/compaction as well as the intermolecular self-association (aggregation/precipitation) of chromatin. The presence of multivalent cations and/or high monovalent salt screens repulsion between fibers and enables positively charged flexible N-terminal tails to mediate attraction between negatively charged chromatin fibers. During chromatin fiber condensation, which was investigated with the much-studied nucleosome arrays reconstituted with Widom 601 DNA templates, two processes occur as a function of increasing salt/divalent cation addition. First, the internal ‘monomolecular’ folding of the array into ‘30 nm fibers’ occurs, followed by intermolecular aggregation (self-association), resulting in the precipitation of chromatin aggregates. The first process was usually monitored via analytical ultracentrifugation (AUC) [32,33]; the latter one via a precipitation assay (PA) (also termed differential centrifugation assay) [31,32,34,35]. In spite of the decades-long in vitro studies of chromatin fiber intermolecular precipitation/aggregation, detailed pictures of the supra-molecular structures and properties of the chromatin aggregates have not been established.

During the last ten years, the formation of various cellular components (proteins, nucleic acids, and their complexes) into droplet-like condensates has received much attention [36,37,38,39,40]. It is generally believed that such condensates play an important role in the membrane-less compartmentalization and concentration of important functional biopolymers. Many of these condensates form by a process called liquid–liquid phase separation (LLPS), which may be considered a free energy minimum state with the formation of condensates dispersed in and in equilibrium with the liquid phase [41]. Recently, Gibson et al. [42] demonstrated that the well-known chromatin fiber self-association aggregation detected using a precipitation (differential centrifugation) assay, when considered at the supramolecular level, may result in droplet condensates by LLPS. It was shown that the process is driven by ionic interactions with the crucial contribution from the histone N-terminal tails. In other words, chromatin fiber self-association, which has been experimentally studied during the last 40 years [35], may result in droplet formation driven by LLPS [13]. In a recent study by Strickfaden and co-workers [43], the picture of the formation of chromatin droplets, induced by multivalent cation and/or high salt, was somewhat questioned. On the basis of their data, these authors suggested that in vitro (and in vivo), the associated chromatin fiber condensates display a solid- or gel-like form and the chromatin droplets were only observed with the presence of BSA in the buffer. It may be noted that in both the work of Gibson et al. [42] and Strickfaden et al. [43], rather short nucleosome arrays (comprising 12 repeats and with variable NRLs) based on the Widom 601 high-affinity nucleosome positioning sequence were used. The generalization of those results to the behavior of large, near-megabase-size chromatin characteristic of TADs in the cell nucleus is therefore not clear. 

In this work, we have investigated the detailed effects of several solvent conditions (with and without BSA), ionic conditions, as well as chromatin fiber length, on the LLPS of chromatin in vitro, forming liquid or solid- or gel-like condensates. We performed comprehensive studies using nucleosome arrays formed with 15 repeats of the 197 NRL (50 bp linker DNA) Widom 601 DNA template (15-197-601) (~3 kbp). In addition, the effect of fiber length on chromatin LLPS was elucidated via additional characterization on nucleosome arrays reconstituted from 62 repeats of 202 bp NRL Widom 601 DNA array template (62-202-601) (~12.5 kbp) and lambda DNA (~48.5 kbp). 

## 2. Materials and Methods

### 2.1. Overexpression and Purification of Histones

*Homo sapiens* histone H2A, H2B, H3 and H4 were overexpressed and purified following published protocols [34,44,45,46,47,48,49]. pET-3a plasmids containing the histone genes were transformed and overexpressed in *E. coli* BL21 (DE3) pLysS cells in 2X TY media. Crude histones were purified via a 26/60 Sephacryl S-200 column (GE Healthcare, MA, USA) followed by a Resource S cation-exchange column (GE Healthcare, MA, USA). 

### 2.2. Preparation of Histone Mutants

Site-specific mutagenesis was conducted on wild-type H3 to generate the H3_C96, 110A mutant and on wildtype H2B to generate the H2B_T116C mutant. The overexpression and purification of H2B_T116C and H3_C96,110A were similar to those of wild-type histones, except that H2B_T116C was overexpressed in *E. coli* BL21 (DE3) cell strain.

### 2.3. Fluorescent Labeling of Histone H2B_T116C

To label H2B_T116C with Alexa Fluro488 or Alexa Fluro647, lyophilized H2B_T116C was dissolved in 20 mM Tris⋅HCl (pH 7.5), 150 mM NaCl; then, 100 mM TCEP was added to the solution with a final concentration of 1 mM and the mixture was incubated at room temperature for 1 h to have cysteines fully reduced, which was followed by buffer exchange to 1X PBS buffer using a 5 mL of HiTrap Desalting column (GE Healthcare). The labeling reaction was conducted via the addition of 1.5 molar excess Alexa Fluor 488 (AF488)-C5-maleimide or Alexa Fluor 647 (AF647)-C2-maleimide into the reduced histone H2B_T116C, followed by incubation in the dark for 4 h at room temperature. The reaction was quenched by adding 10 mM DTT. Then, the AF488-labeled or AF647-labeled histone H2B was purified using a 5 mL HiTrap Desalting column in storage buffer (20 mM Tris⋅HCl pH 7.5, 150 mM NaCl and 1 mM DTT), and pure fractions were pooled and dialyzed against 5 mM b-mercaptoethanol in MilliQ H_2_O.

### 2.4. Histone Octamer Refolding

Histones (H2A: H2B: H3: H4) were mixed in unfolding buffer (7 M Gdn·HCl, 10 mM Tris⋅HCl, 10 mM DTT, pH 7.5) at a molar ratio of 1.2:1.2:1:1, and the mixture was dialyzed against refolding buffer (10 mM Tris⋅HCl, 2 M NaCl, 1 mM EDTA, 5 mM beta-mercaptoethanol, pH 7.5) overnight. The HO was purified via gel filtration chromatography using a HiLoad 16/600 Superdex 200 pg column (GE Healthcare, MA, USA), and fractions were checked with 18% SDS-PAGE.

The refolding of octamer with AF488- or AF647-labeled H2B follows the same protocol except that the non-labeled H2B was substituted with 100% labeled H2B histone, and wild-type H3 was substituted with H3_C96, 110A. The quantity of purified H2B-labeled HO was calculated by measuring absorbance at 280, 495 and 633 nm with the molar extinction coefficients of HO, AF488 and AF647 setting to be 44,700/M⋅cm, 72,000/M⋅cm and 265,000/M⋅cm, respectively. Then, 100% labeled histone octamers were confirmed through the presence of 2:1 stoichiometric excess of fluor to histone octamer.

### 2.5. DNA Preparation

The 15-197-601 DNA (15 repeats of the Widom 601 high-affinity nucleosome positioning sequence with 197 bp NRL) originally constructed in pUC18 vector from Daniela Rhodes laboratory was subcloned to pWM530 vector. The amplified plasmids were digested by EcoRV, followed by PEG6000 fractionation. The desired DNA fragment was further purified via a HiPrep 26/60 Sephacryl S-500 HR column (GE Healthcare, MA, USA). 

The 62-202-601 DNA (62 repeats of the Widom 601 high-affinity nucleosome positioning sequence with 202 bp NRL) originally constructed in pETcoco vector from Daniela Rhodes laboratory was subcloned to pUC18 vector. The amplified plasmids were digested using EcoRV, DraI and HaeII, followed by PEG6000 fractionation. The desired DNA fragment was further purified via a Sephacryl S-1000 column. The lambda DNA (48,502 bp) were purchased from New England Biolabs (N3011L).

### 2.6. Chromatin Preparation

Reconstitution of nucleosome arrays was conducted following the published protocols [34,45,46,47,48,49]. For the 15-197-601 array, 15-197-601 DNA, purified HOs, and 147-bp (or 157-bp) competitor DNA were mixed and were subjected to salt gradient dialysis at 4 °C overnight in TEN 2.0 buffer (10 mM Tris⋅HCl, 2 M NaCl, and 0.1 mM EDTA, pH 7.5) to TEN 0.01 buffer (10 mM Tris⋅HCl, 10 mM NaCl, and 0.1 mM EDTA, pH 7.5). The mass ratio of 15-197-601 DNA and 147-bp (or 157-bp) competitor DNA was 1:0.05 for all reconstitutions. The array solution was centrifuged at 20,000× *g* for 10 min after dialysis. The saturation point of reconstituted arrays was determined via electrophoresis in 0.8–1.1% agarose gel. In the preparation of 62-202-601 nucleosome array, a trace amount of backbone digestion fragments in the DNA stock served as the competitor DNA in the reconstitution. The lambda DNA array preparation was similar but without the addition of competitor DNA, and it exactly followed and was carried out with the same materials as in our recent work [48]. The preparation of AF488- and AF647-labeled nucleosome arrays followed the same protocol, except that the HO used were 5% AF488- or AF647-labeled, and the salt dialysis processes were conducted in the dark.

Protocols of high-quality reconstitution have been well established in much of the recent work from our [46,47,48,49] and other laboratories [50,51,52,53]. To ensure the optimal stoichiometry of the reconstituted nucleosome arrays, small-scale reconstitutions from a batch of HO and DNA templates with varying molar ratios were performed. The reconstitution products were typically analyzed via gel electrophoresis, in combination with AUC and digestion with restriction enzymes when applicable. Figure S1 demonstrates an example of assessing the small-scale 15-197-601 nucleosome array reconstitution products by AUC and AvaI digestion. Figure S2A shows the agarose gel electrophoresis data for a set of large-scale reconstitution products used for the following confocal microscopy investigation, suggesting the high quality of the reconstituted 15-197-601 and 62-202-601 nucleosome arrays. It is noted that, in comparison with the products from small-scale reconstitution, no competitor DNA was detected, which is attributed to the extra concentration or buffer exchange processes using a membrane concentrator (50 kDa cutoff), leading to small DNA fragments passing through the membrane. This further eliminated the possible contamination of the final samples utilized for phase separation experiments. The purity of 15-197-601 and 62-202-601 nucleosome arrays for phase separation characterization were further checked via gel electrophoresis analysis of supernatants from Mg^2+^ precipitation assays. As illustrated in Figure S3, no DNA or proteins were detected in the supernatants when the nucleosome arrays were fully precipitated by adding Mg^2+^ and applying centrifugation. Furthermore, the nucleosome arrays reconstituted from lambda DNA were performed following and in parallel to that of our recent paper, where the arrays were visualized with negative stain electron microscopy [48], and this resulted in an average nucleosome occupancy of about one HO per 200 bp DNA. An example of the gel electrophoresis of small-scale reconstitution of lambda nucleosome arrays is provided in Figure S4, where the molar ratio of HO per 200 bp lambda DNA was set to 1.0 (based on defining 1.0 as one HO per 200 bp lambda DNA) for large-scale reconstitution. Taken together, the sample quality controls and assessments ensure the saturation and integrity of the nucleosome arrays used in the phase separation experiments. 

### 2.7. Preparation of 384-Well Microscopy Plates

Before adding nucleosome array samples for microscopy experiments, the 384-well microscopy plates were pre-treated. The microscopy plates with high-performance #1.5 cover glass (product No. P384-1.5H-N, Cellvis) were treated with 5% Hellmanex at 37 °C for 4 h and rinsed with MilliQ water. Subsequently, the plates were treated with 1 M NaOH for 1 h and were rinsed with MilliQ water, followed by incubation with 25 mg/mL 5K mPEG-silane (PEGWorks) in 95% ethanol overnight at room temperature. The plates were then rinsed with 95% ethanol and MilliQ water before being dried up and sealed with adhesive PCR plate foils (Thermo). 

Right before the phase separation experiments, the foil was removed, and then, glasses of the microscopy plates were passivated by 100 mg/mL BSA for 30 min. Upon being rinsed with MilliQ water, the microscopy plates were ready for the samples to be added in immediately.

### 2.8. Preparation of Phase Separation Samples

Nucleosome array samples in TEN 0.01 buffer were dialyzed against chromatin dilution buffer at 4 °C overnight. To prepare the phase separation samples, nucleosome arrays were mixed with chromatin dilution buffer and staining dye to achieve the designated array concentrations. For each sample, an equal volume of phase separation buffer was added into the solution without mixing, and then the sample was incubated at room temperature for 30 min in the dark before it was mixed and transferred to the PEGylated and passivated microscopy plates. The microscopy wells were sealed with transparent scotch tape and ready for microscopy imaging. Details of different phase separation buffers are listed in Table S1.

### 2.9. Two-Color Mixing Assay

For the two-color mixing assay, reconstituted nucleosome arrays harboring AF488- or AF647-labeled 15-197 in TEN 0.01 buffer were mixed with chromatin dilution buffer to achieve the desired array concentration, and then, an equal volume of phase separation buffer was added into the solution that was incubated for 30 min before being gently mixed via pipetting. An equal volume of AF488-labeled array and AF647-labeled 15-197 array was mixed and incubated for another 20 min and then transferred to the PEGylated and passivated wells for microscopy imaging.

### 2.10. Confocal Fluorescence Microscopy 

Confocal fluorescence microscopy experiments were performed with a Zeiss LSM 710 or 800 confocal microscope system. Non-fluorescent nucleosome arrays were stained with DyeCycle stain (ThermoFisher). Different levels of DyeCycle were first tested to choose the optimal concentration for array staining. The confocal microscopy images were collected within 30–60 min after the last step of sample incubation to eliminate the condensate aging effects. At least four images were collected for each sample in the phase separation experiments. In addition, biological replicates were prepared and characterized via confocal fluorescence microscopy under several conditions, confirming the reproducibility of the phase separation characterization (Figure S5). The raw data were exported to tiff images before further processing. The area percentages of condensates were obtained by analyzing the images using the ImageJ software. 

## 3. Results and Discussion

### 3.1. Chromatin Droplet Formation by LLPS Depends on Ionic Conditions

**Figure 1:**
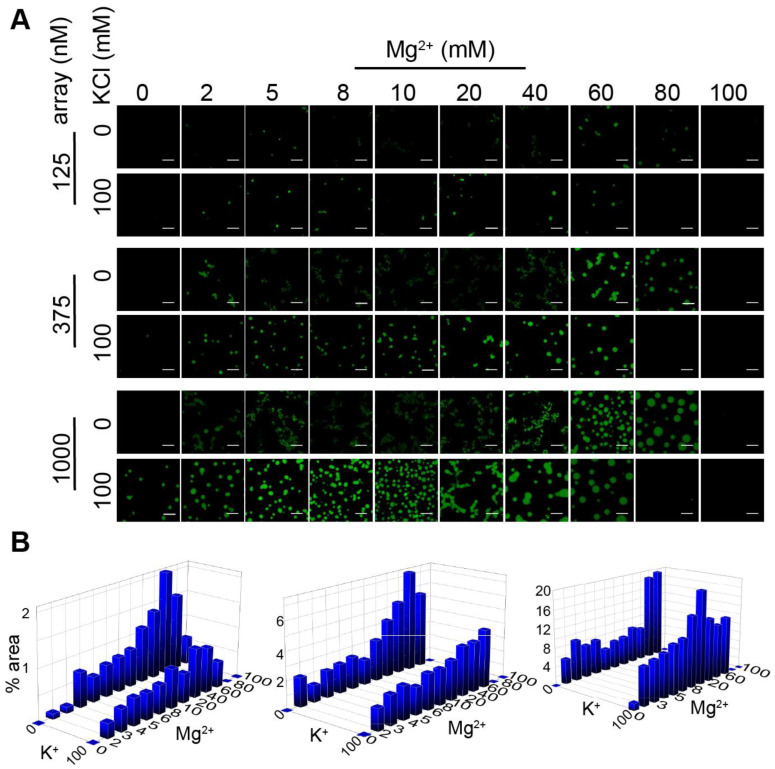
Reconstituted nucleosome arrays undergo LLPS in a salt-dependent manner. (**A**) Confocal fluorescence microscopy images of 15-197-601 nucleosome arrays with DNA stained by DyeCycle in the absence and presence of 100 mM KCl at various MgCl_2_ and nucleosome array concentrations. Images for samples in conditions with wider range of MgCl_2_ concentrations are shown in Figure S1. (**B**) Quantified percentages of fluorescent condensate areas in the images as a function of KCl and MgCl_2_ concentrations for nucleosomes arrays with a concentration of 125 nM (**left**), 375 nM (**middle**) and 1000 nM (**right**). Scale bar represents 10 μm.

First, we investigated the aggregation and droplet formation of in vitro reconstituted nucleosome arrays (15-197-601) at varying concentrations of chromatin, KCl and MgCl_2_ (Figure 1). The chromatin concentration effects were explored by experiments with three 15-197-601 array concentrations. Several studies utilized the condensate-occupying areas to qualitatively evaluate the LLPS behaviors of MeCP2 or HP1 interacting with nucleosome arrays [54,55]. Here, employing the same approach, confocal fluorescence microscopy data demonstrated that an increased nucleosome array concentration promotes the formation of condensate, as evidenced by condensate-occupying areas in the solution. For example, the percentages of nucleosome array condensate area could reach nearly 2%, 6% and 20% maximum with an array concentration of 125, 375 and 1000 nM, respectively, in the presence of Mg^2+^ and the absence of KCl. The appearance of the condensates varies significantly with ion concentrations. The presence of a physiological concentration of KCl (100 mM) leads to the formation of droplets formed by LLPS as further suggested by the two-color mixing assays that are discussed below (Section 3.3). In the absence of added KCl, the condensates generally appear gel-like or fibrous, suggesting solid-like aggregation, which can be clearly observed in the images of samples containing 0–40 mM MgCl_2_ and no added KCl with an array concentration of 375 nM or 1000 nM (Figure 1A). Increasing KCl concentration enhances the fluidity of the condensates and leads to transiting between different phases at critical conditions (Figure S7). For example, when the Mg^2+^ concentration is 20 mM or less, the gel- or solid-like aggregates transition to spherical droplets with increasing sizes when the KCl concentration is increased to 200 mM. When the Mg^2+^ concentrations are between 40 and 80 mM, the condensates are gradually dissolved with an increasing amount of KCl. Overall, KCl is necessary to promote the formation of more well-defined and larger droplets. It is well known that Mg^2+^ can modulate the aggregation of nucleosomes. Here, we observed that with a higher MgCl_2_ concentration, the nucleosome arrays initially form spherical-like droplets, and then, the condensates appear as more solid-like aggregates in the absence of KCl (Figure 1 and Figure S6). When the Mg^2+^ concentration reaches beyond a critical concentration, condensates again become more spherical-like droplets, and finally, at high Mg^2+^ concentrations, they re-solubilize without precipitation [29,30,31]. In the presence of KCl (100 and 200 mM), the same phenomenon was observed, except there was no stage of gel- or solid-like aggregates because of the enhanced fluidity induced by additional KCl. The general observation is that the formation of droplets, which implies an LLPS mechanism, requires the presence of a physiological concentration of salt. The results are in general agreement with those of Gibson et al. [42]. It is noted that the condensates including the droplets form pellets following centrifugation, which is demonstrated by the precipitation assay characterization (Figure S6). Overall, Mg^2+^ prompts the aggregation of the 15-197-601 nucleosomes array, and adding KCl with a near-physiological concentration softens the aggregates and leads to liquid droplet formation with high fluidity when the critical concentrations are reached, which is further demonstrated by the two-color assays discussed below.

### 3.2. Chromatin Droplet Formation in Different Buffer Conditions

**Figure 2:**
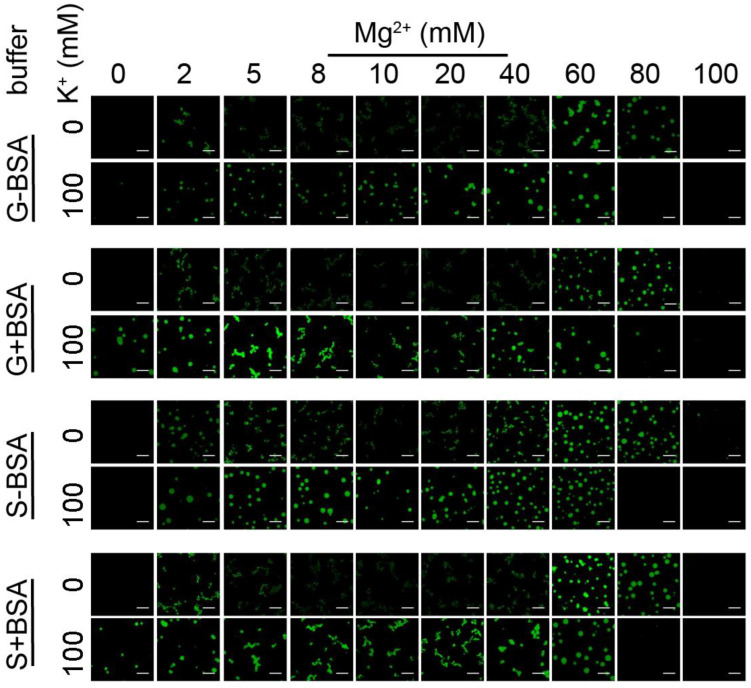
Reconstituted nucleosome arrays undergo LLPS in different buffer conditions. Confocal fluorescence microscopy images of 15-197-601 nucleosome arrays with DNA stained by DyeCycle in the absence and presence of 100 mM K^+^ at four buffer conditions as a function of Mg^2+^ concentration. The nucleosome array concentration is 375 nM. The detailed buffer components are listed in Table S1. Images for samples in conditions with wider range of Mg^2+^ concentrations are shown in Figure S8. Scale bar represents 10 μm.

We next compared the buffer conditions with or without the presence of reduced BSA and acetate anion in the solution, which was suggested by Strickfaden et al. [43] to be a necessary condition for chromatin droplet formation that is characteristic of a LLPS mechanism (Figure 2). We chose buffer conditions that were used to study nucleosome arrays in LLPS by Gibson et al. [42] and by Strickfaden et al. [43], respectively, adding or eliminating BSA to generate four buffer systems in total (Table S1). The confocal fluorescence microscopy data for 15-197-601 nucleosome arrays suggest that the buffer conditions with or without BSA/acetate yield largely similar results and lead to droplet formation under a range of MgCl_2_ salt conditions (see the images in the range 0–4 mM Mg^2+^ in Figure 2 and Figure S8), although there is some tendency for the presence of BSA/acetate to result in more solid-like fibrous aggregates at intermediate Mg^2+^ concentrations (5–20 mM). However, the general picture discussed above prevails, namely that the presence of a physiological K^+^ concentration is necessary to promote droplet formation, while the absence of a high K^+^ concentration tends to result in solid-like fibrous aggregates. In the present study, all the experiments were performed in buffers with the absence of BSA/acetate (except for the comparisons with such conditions).

### 3.3. Two-Color Mixing Assay Reveals the Liquid-Like Properties of Chromatin Condensates in the Presence of Physiological KCl

**Figure 3:**
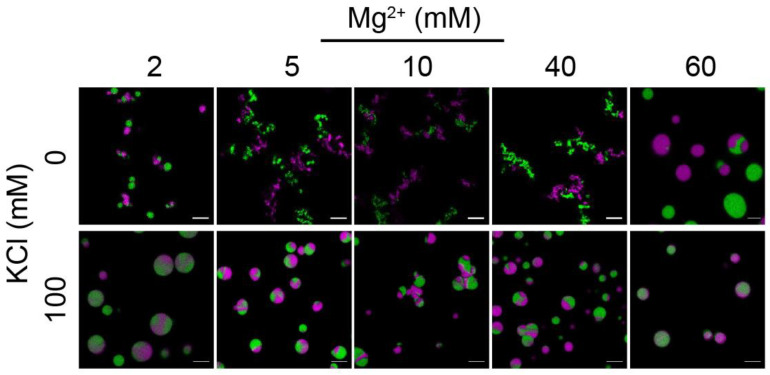
Two-color mixing assay characterization of the phase separation states of 15-197-601 nucleosome arrays. Confocal fluorescence microscopy images of samples at conditions with various KCl and MgCl_2_ concentrations are shown. Nucleosomes arrays containing histone H2B T116C labeled with AF488 (green) or AF647 (purple) were treated with phase separation buffer separately and were incubated for 30 min before mixing. Then, the mixture was incubated for another 20 min before imaging. The nucleosome array concentration is 375 nM. Scale bar represents 5 μm.

We further characterized the dynamic and liquid-like properties of the chromatin condensates using a two-color mixing assay. Histone H2B T116C containing AF488 (green) or AF647 (purple) were used to prepare labeled 15-197-601 nucleosome arrays. The nucleosome arrays with single fluor labeling were treated with the phase separation buffer separately and were incubated for 30 min before mixing. The mixed solutions were incubated for another 20 min before confocal fluorescence microscopy characterization. As displayed in Figure 3, under the conditions without KCl, adding Mg^2+^ with concentrations of about 2–40 mM results in the formation of phase-separated precipitates, which are characterized by gel-like fibrous aggregates and show no mixing or fusion over time. When the Mg^2+^ concentration is higher, near-spherical droplets are observed, showing considerable fluidity, as evidenced by the fusion of a number of particles. Differently, the condensates formed at 100 mM KCl and various Mg^2+^ concentrations are generally fused, as indicated by the overlaid fluorescent signals of an individual droplet. This suggests that under those conditions, the chromatin condensates form spherical dynamic droplets (contrary to the fiber state in the absence of 100 mM KCl), and the individual arrays exchange between droplets under the timescale of the experiment. Experiments performed in the same buffer but with the addition of BSA/acetate displayed similar results (Figure S9), although a slight tendency for solid-like precipitates was seen in the presence of 100 mM KOAc at intermediate Mg^2+^ concentrations.

### 3.4. The Formation of Chromatin Droplets Depends on Nucleosome Array Fiber Length

**Figure 4:**
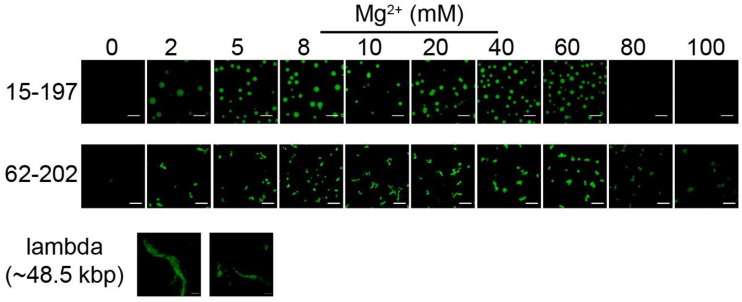
Reconstituted nucleosome array LLPS depends on chromatin fiber length. Confocal fluorescence microscopy images of nucleosome arrays reconstituted using 15-197-601, 62-202-601, lambda DNA (~48.5 kbp). Images of 15-197-601 and 62-202-601 nucleosome arrays were taken for samples in the presence of 100 mM KCl and various Mg^2+^ concentrations. Images for lambda DNA nucleosome arrays were taken for samples at 0 mM (left) and 100 mM (right) KCl and in the absence of Mg^2+^, where solid-like condensates were observed (same as in the presence of Mg^2+^). The nucleosome array concentrations are 375 nM. Scale bar represents 10 μm. Images for samples at conditions with wider range of Mg^2+^ concentrations are shown in Figure S10.

**Figure 5:**
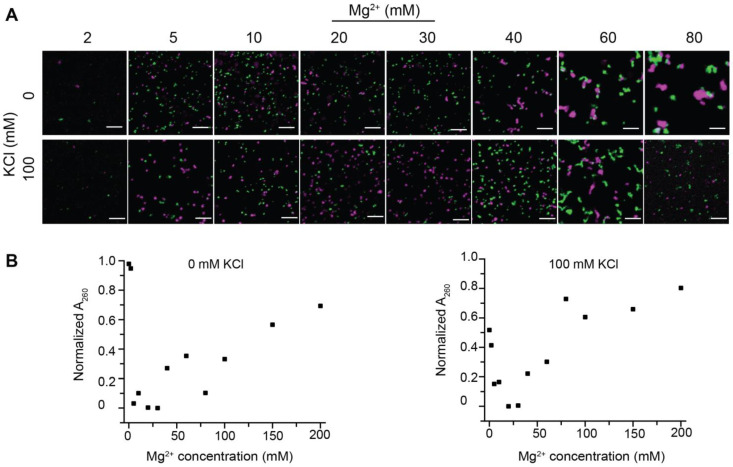
Two-color mixing assay characterization of the solid-like aggregation of 62-202-601 nucleosome arrays. (**A**) Confocal fluorescence microscopy images of samples at conditions with various KCl and MgCl_2_ concentrations are displayed. Nucleosome arrays containing histone H2B T116C labeled with AF488 (green) or AF647 (purple) are treated with phase separation buffer separately and were incubated for 30 min before mixing. Then, the mixture was incubated for another 20 min before imaging. The array concentration is 375 nM. (**B**) Precipitation assay characterization of 62-202-601 nucleosome arrays. The percentage of arrays in the supernatant after centrifugation (10,000× *g* for 5 min) are calculated by the normalized absorbance at 260 nm. Scale bar represents 5 μm.

In the previous studies of chromatin LLPS [42,43], rather short nucleosome arrays comprising 12 repeats of the Widom 601 nucleosome positioning sequence and with variable NRLs in the range of 172–207 bp were used in the in vitro phase separation studies. It may not be straightforward to carry over such results to the physiological relevance in vivo, where chromatin is organized in various hierarchical levels of different length scales of chromatin domains of the chromosomes. For example, TADs are generally composed of up to megabase-long chromosomal fragments. In order to investigate the effect of the individual chromatin fiber (nucleosome arrays) length, we compared the results for the 15-197-601 arrays (~3 kbp) with arrays formed with 62 repeats of the 202 bp NRL Widom 601 DNA array templates (62-202-601) (~12.5 kbp), and we also characterized the phase separation behavior of nucleosome arrays obtained by reconstitution with lambda DNA (~48.5 kbp) (Figure 4). The results clearly suggest that neither the 62-202-601 nor the lambda DNA nucleosome arrays form spherical droplets indicative of liquid-like states formed by LLPS. In particular, the lambda DNA chromatin fibers appear highly gel-like and immobile, as suggested by the relatively large aggregates formed with or without KCl (Figure 4). The condensates formed by 62-202-601 nucleosome arrays are gel- or solid-like, and we further conducted two-color assay experiments to investigate their fluidity. As illustrated in Figure 5A, the condensates formed by the 62-202-601 nucleosome arrays with or without KCl at a wide range of Mg^2+^ concentrations are gel- or solid-like, and the individual array molecules do not exchange between aggregates. Similar condensates are observed for the conditions with 100 mM KCl; meanwhile, the presence of KCl leads to a lower degree of aggregation at a given Mg^2+^ concentration in comparison with the conditions without KCl, again suggesting that KCl attenuates aggregation and enhances the mobility/fluidity of the condensates. The precipitation assay performed for 62-202-601 nucleosome arrays shows that increasing Mg^2+^ concentration further leads to re-solubilization of the arrays, where the percentages of arrays that form pellets are generally higher than those of 15-197-601 nucleosome arrays at a given Mg^2+^ concentration (Figure 5B and Figure S6B). We also conducted two-color and precipitation assays for the 62-202-601 nucleosome arrays in conditions including BSA/acetate, and similar results were obtained (Figure S11). In addition, the BSA/acetate-containing buffer system shifted the re-solubilization of the precipitates to conditions of a higher Mg^2+^ concentration threshold (Figure 5B and Figure S11). 

A recent study reported that nucleosome arrays with (10n + 5) bp linker spacing favor LLPS in comparison with those having 10n linker spacing [42]. In the present work, it is demonstrated that the 62-202-601 nucleosome array (55 bp linker) does not undergo LLPS process to form dynamic droplets under the tested conditions, instead precipitating to solid- or gel-like states, while 15-197-601 nucleosome arrays (50 bp linker) do form dynamic droplets under most tested conditions. Therefore, even though the 62-202-601 nucleosome array has (10n + 5) bp linker spacing that would promote droplet formation according to Gibson et al. [42], no dynamic droplet formation is observed for these (10n + 5) bp spacer arrays in the current work. The 15-197-601 nucleosome arrays, which possess 10n linker spacing and are less prone to droplet formation, still exhibit LLPS behavior, as demonstrated in our experiments. Thus, the current results, including those for the even longer lambda DNA arrays, together with the observations by Gibson et al. [42], further support the concept that nucleosome arrays with longer lengths disfavor dynamic droplet formation. The origin of the absence of dynamic droplet formation for 62-202-601 is likely due to the long fiber length.

## 4. Conclusions

Our results for the reconstituted 15-197-601 nucleosome arrays are generally in line with observations reported by two recent studies [42,43] and demonstrate the liquid-like properties of chromatin condensates formed under varying ionic conditions. The characterization highlights the importance of the presence of a physiological amount of KCl for the formation of these condensates [42]. Under the conditions that we used, gel- or solid-like precipitates are generally observed in the absence of 100 mM KCl. 

**Figure 6:**
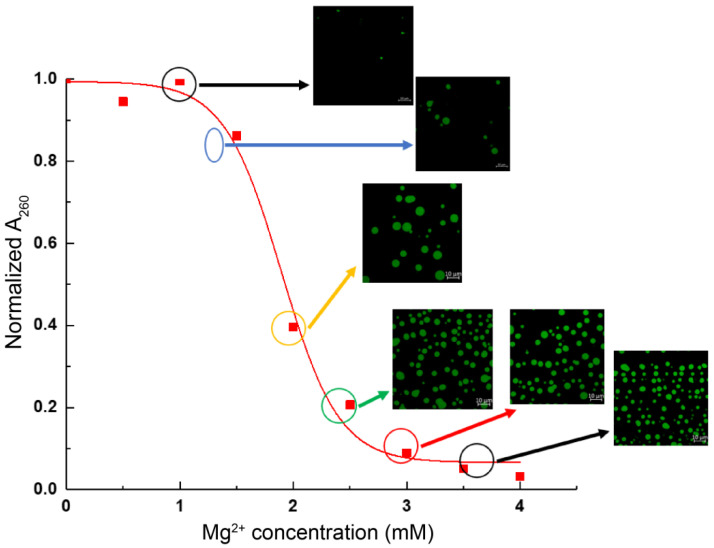
Precipitation assay curve and confocal fluorescence microscopy characterization of nucleosome arrays as a function of Mg^2+^ concentration. DyeCycle was used to stain the DNA of the nucleosome arrays. The KCl concentrations in the buffers are 75 mM.

As discussed in the lucid review by Hansen et al. [35], precipitation assay (also termed differential centrifugation assay) studies of in vitro reconstituted nucleosome arrays have been conducted for decades in order to investigate the intermolecular chromatin fiber–fiber association/aggregation and how it depends on various solution conditions, as well as on the properties of the chromatin, e.g., histone tail modifications. An example of a precipitation assay curve is displayed in Figure 6 and was obtained in our present study, showing the concentration of array remaining in the supernatant following centrifugation, where the precipitates form pellets. The microscopy images are displayed for the nucleosome arrays at the corresponding buffer conditions. These illustrate that the material state of the precipitate is characterized by droplets formed by the LLPS mechanism under those conditions. 

In this work, we demonstrated that relatively shorter nucleosome arrays, 15-197-601, undergo robust LLPS and form droplets with high fluidity at physiologically relevant ion concentrations. In contrast, as illustrated by experiments on 62-202-601 and lambda nucleosome arrays, longer nucleosome fibers tend to form gel- or solid-like aggregates under the same conditions. Thus, the phase separation behaviors and the properties of chromatin condensates are strongly affected by the chromatin fiber length and ion conditions. It may be noted that the in vitro reconstituted lambda DNA chromatin, which was characterized in detail in our recent work, forms nucleosome clutches [48]. This could suggest that the physical states of chromatin may also correlate with the sizes of such nucleosome clutches in vivo. Chromatin condensation/aggregation can be modulated by a variety of factors, of which we only delineate the effects of some in this work. For example, H1 is an essential protein to condensate and stabilize chromatin [56,57,58] and has been shown to modulate the phase behaviors of chromatin or DNA [42,59]. Such comprehensive characterization of the phase separation behaviors of chromatin and effector proteins, both in vivo and in vitro, requires future studies to fully understand the physical states and phase transitions of chromatin, and its functional significance. 

## Supplementary Materials

The following supporting information can be downloaded at: https://www.mdpi.com/2073-4409/11/19/3145/s1.
