# Supplementary material for: Chromatin Liquid–Liquid Phase Separation (LLPS) Is Regulated by Ionic Conditions and Fiber Length
Source: Cells. 2022 Oct 6;11(19):3145. doi: 10.3390/cells11193145 (PMC9564186; doi:10.3390/cells11193145)
Supplement: Supplementary file 1 [file cells-11-03145-s001.zip › Supplementary Material_Final.pdf]

Supplementary Material

Chromatin Liquid–Liquid Phase Separation (LLPS) Is Regulated by Ionic Conditions and Fiber Length

Qinming Chen [1,†], Lei Zhao [2,†], Aghil Soman [1], Anastasia Yu Arkhipova [2,3], Jindi Li [2], Hao Li [2], Yinglu Chen [2], Xiangyan Shi [2,*] and Lars Nordenskiöld [1,*]

[1] School of Biological Sciences, Nanyang Technological University, Singapore 637551, Singapore
[2] Department of Biology, Shenzhen MSU-BIT University, Shenzhen 518172, China;
[3] Biological Faculty, Lomonosov Moscow State University, 119991 Moscow, Russia
* Correspondence: xyshi@smbu.edu.cn (X.S.); LarsNor@ntu.edu.sg (L.N.)
† These authors contributed equally to this work.

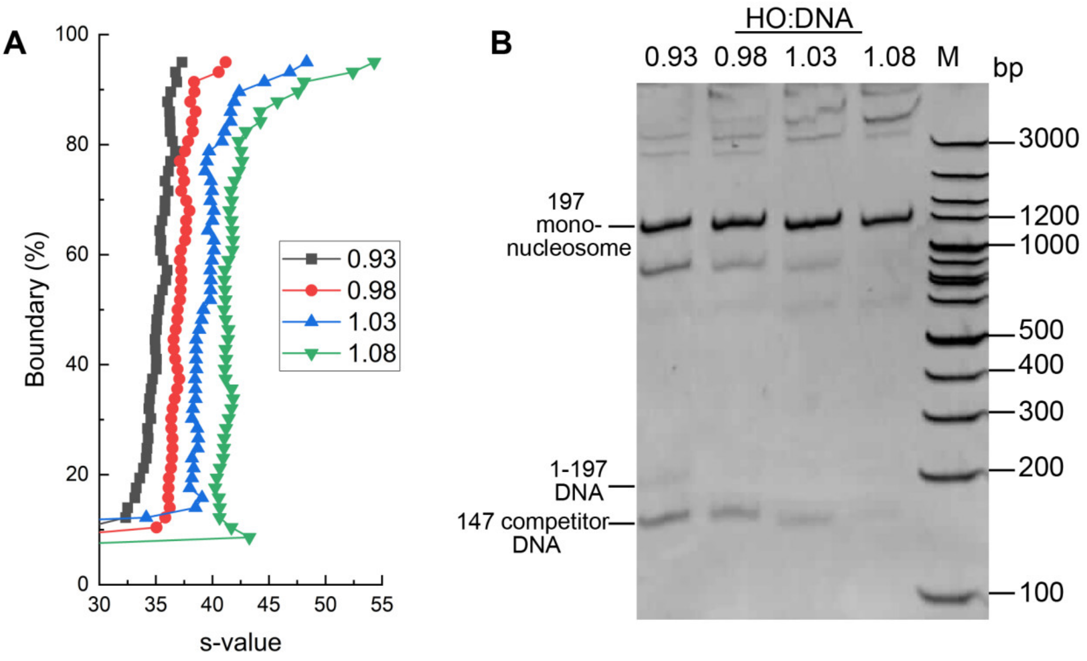

Figure S1. Optimization for 15-197-601 nucleosome array reconstitution. (A) van Holde-Weischet curves showing the boundary distribution curves for 15-197-601 nucleosome arrays reconstitution products using HO and 15-197-601 DNA repeat with the (HO:DNA repeat unit) molar ratios of 0.93, 0.98, 1.03 and 1.08. The analytical ultracentrifugation sedimentation velocity (AUC-SV) experiments were conducted using a Beckman XL-A/XL-I analytical ultracentrifuge (Beckman Coulter, Brea, CA) with an 8-hole An-50 Ti analytical rotor. (B) 0.8% agarose gel images for the AvaI digestion of the 15-197-601 nucleosome arrays reconstituted from the HO and 15-197-601 DNA templates with the (HO:DNA repeat unit) molar ratios of 0.93, 0.98, 1.03 and 1.08. “M” denotes the lane of DNA marker. The bands in the sample lanes above 3000 bp corresponding to tri- and di-nucleosomes. It is illustrated that the competitor DNA at the ratio of 1.08 was completely consumed. There was some precipitation as evidenced from the nanodrop reading. Thus, overall, the (HO:DNA repeat) molar ratio of 1.05 for was used for the large-scale reconstitution for this batch of materials.

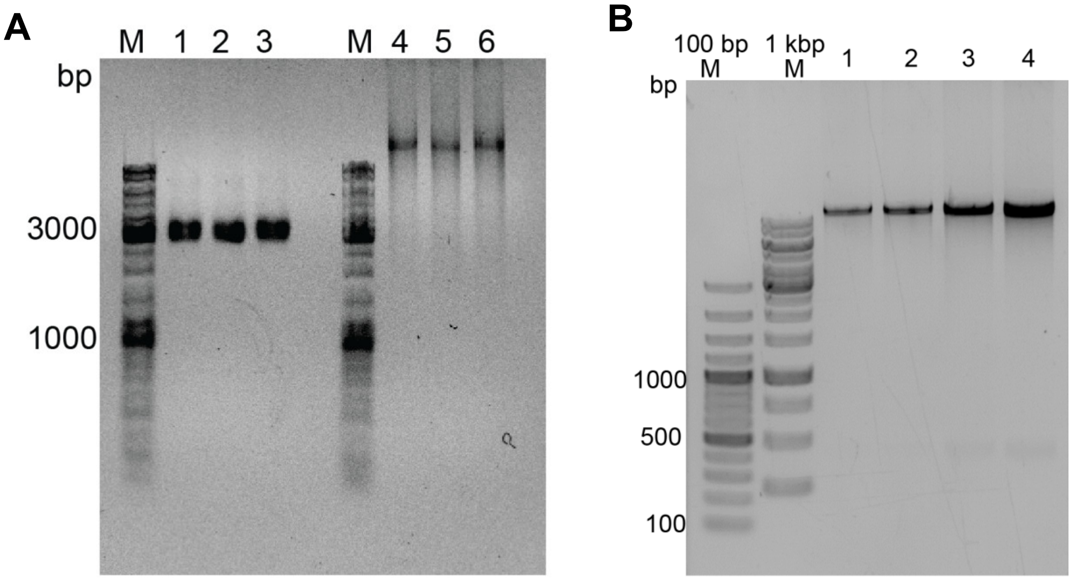

Figure S2. (A) 1% agarose gel electrophoresis images for the 15-197-601 and 62-202-601 nucleosome arrays. Lane 1-3 are 15-197-601 nucleosome arrays that harbor WT, Alexa Fluro488 and Alexa Fluro647 labeled H2B, respectively. Lane 4-6 are 62-202-601 nucleosome arrays that harbor WT, Alexa Fluro488 and Alexa Fluro647 labeled H2B, respectively. (B) 0.8% agarose gel electrophoresis images for the 62-202-601 DNA with loading amounts 16 ng, 32 ng, 48 ng and 80 ng shown in lane 1-4, respectively. Trace amount of backbone digestion fragments were observed with high loading amounts for this sample, where they can serve as additional competitor DNA in reconstitution. "M" denotes the lanes for DNA marker.

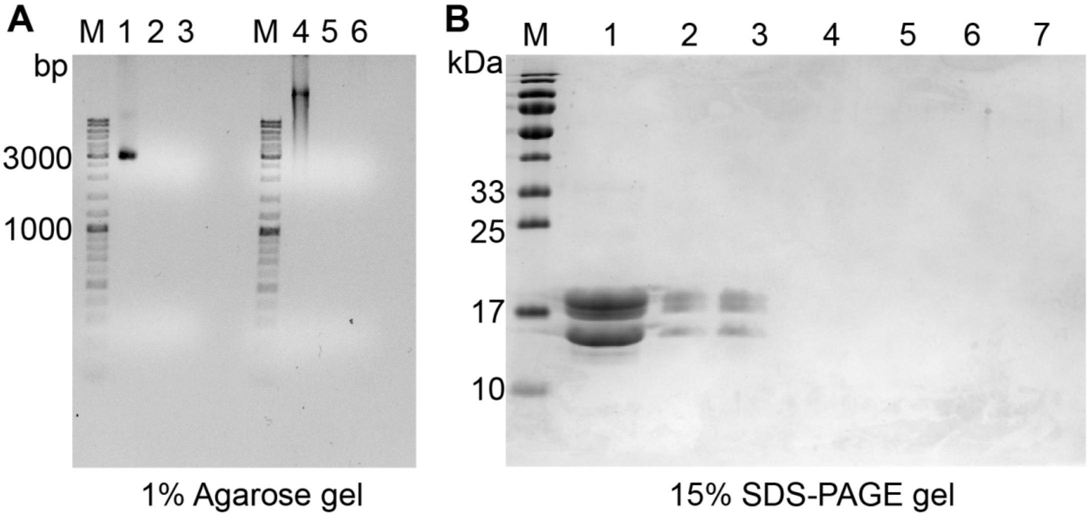

Figure S3. Gel electrophoresis analysis of supernatants from Mg^{2+} precipitation assays performed for 15-197-601 and 62-202-601 nucleosome arrays showing no free DNA or HO contamination in the array samples. (A) 1% agarose gel images for 15-197-601 nucleosome array (lane 1), supernatant of Mg^{2+} precipitated 15-197-601 nucleosome arrays in the presence of 0 and 200 mM KCl (lane 2 and 3), 62-202-601 array (lane 4), supernatant of Mg^{2+} precipitated 62-202-601 arrays in the presence of 0 and 200 mM KCl (lane 5 and 6). "M" denotes the lanes for DNA marker. (B) 15% SDS-PAGE gel for histone octamer (lane 1), 15-197-601 nucleosome array (lane 2), 62-202-601 nucleosome array (lane 3), supernatants of Mg^{2+} precipitated 15-197-601 nucleosome array in the presence of 0 and 200 mM KCl (lane 3 and 4), supernatants of Mg^{2+} precipitated 62-202-601 nucleosome array in the presence of 0 and 200 mM KCl (lane 6 and 7). In the Mg^{2+} precipitation, nucleosome array solutions were added with $MgCl_2$ to reach a series of Mg^{2+} concentrations between 0-200 mM, and the mixtures were centrifuged at 12,000 xg for 5 min and A260 absorbance were measured for the supernatants. Those supernatants having the lowest absorbance for each nucleosome array samples were analyzed by the gel electrophoresis as shown in A and B.

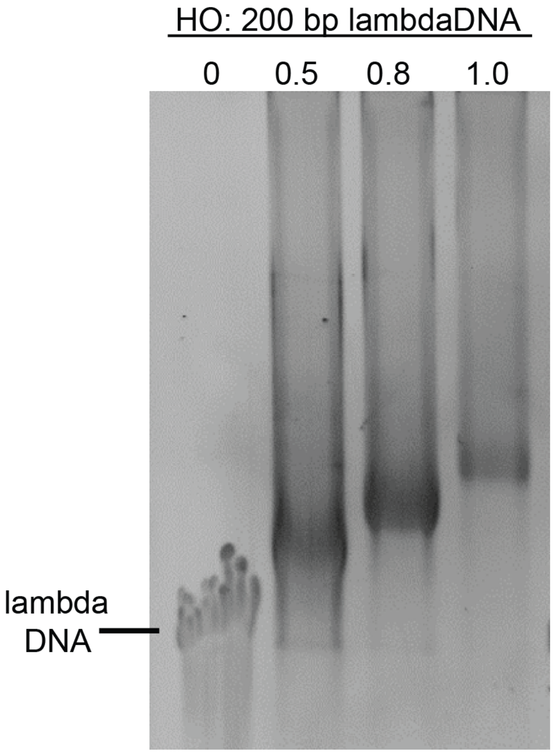

Figure S4. Small-scale reconstitution of lambda nucleosome array for optimizing the molar ratio of histone octamer per 200 bp lambda DNA. The full length of the lambda DNA is 48,502 bp. 1.0 was chosen to use for the large-scale reconstitution of lambda nucleosome array.

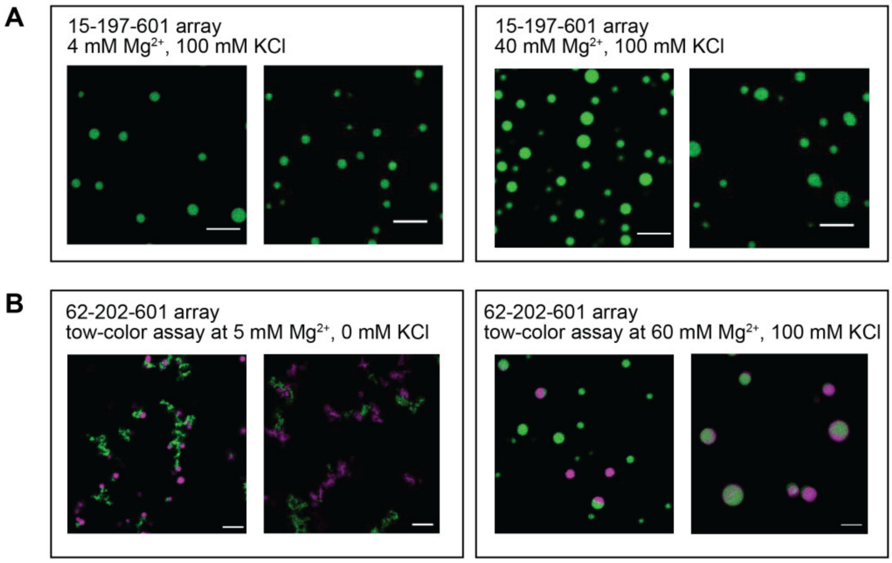

Figure S5. Examples of biological replicates of confocal fluorescence microscopy characterization of nucleosome arrays under several conditions. (A) Confocal fluorescence microscopy images of 15-197-601 nucleosome arrays in the presence of 100 mM KCl at two different $MgCl_2$ concentrations. The nucleosomes array concentration is 375 nM. Scale bar represent 10 µm. For each condition, the images on the right side are presented in Figure 1 or S6, while the images to the left represent data obtained for array samples reconstituted with different batch of refolded HO and on a different instrument. (B) Two-color confocal fluorescence microscopy images of 62-202-601 nucleosome arrays at two conditions. Scale bar represent 5 µm. For each condition, the images on the right side are presented in Figure 3, while the images to the left represent data obtained for array samples reconstituted with different batch of refolded HO and on a different instrument.

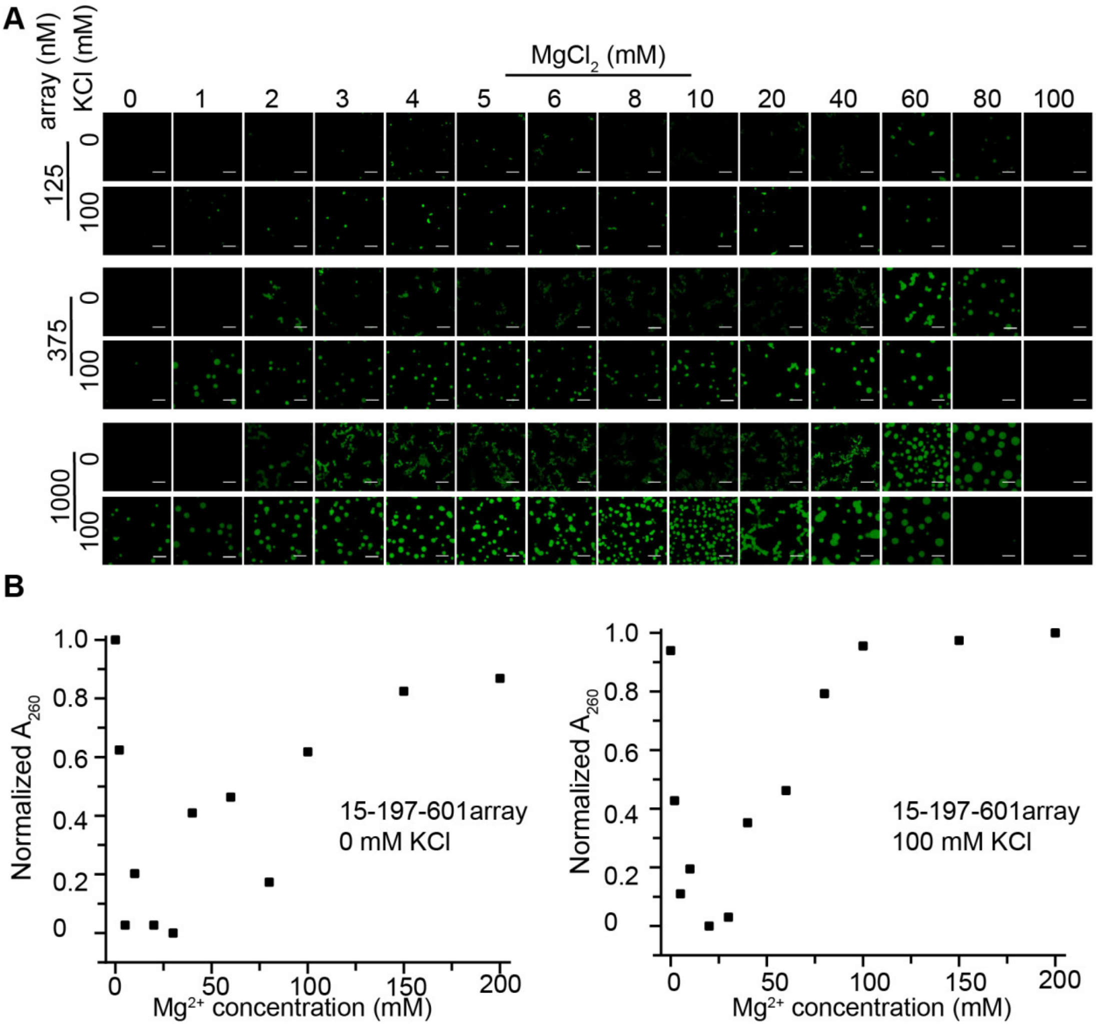

Figure S6. (A) Confocal fluorescence microscopy images of 15-197-601 nucleosome arrays with DNA stained by DyeCycle in the absence and presence of 100 mM KCl at various $MgCl_2$ and nucleosome array concentrations. Scale bar represent 10 µm. (B) Precipitation assay characterization of 15-197-601 nucleosome arrays. The percentage of arrays in the supernatant after centrifugation 10,000 xg for 5 min) are calculated by the normalized absorbance at 260 nm.

Table S1. The components of four buffer systems used in this study.

Buffer system	G-BSA	G+BSA	S-BSA	S+BSA
Tris	25 mM Tris·HCl, pH 7.5	25 mM Tris·OAc, pH 7.5	10 mM Tris·HCl, pH 7.8	10 mM Tris·OAc, pH 7.5
Mg^{2+}	$MgCl_2$	$Mg(OAc)_2$	$MgCl_2$	$Mg(OAc)_2$
K^+	KCl	KOAc	KCl	KOAc
EDTA	0.1 mM	0.1 mM	0.1 mM	0.1 mM
DTT	1 mM	5 mM	1 mM	5 mM
BSA	–	0.1 mg/ml	–	0.1 mg/ml
glycerol	5% w/v	5% w/v	2.5% w/v	2.5% w/v
glucose oxidase	2 µg/ml	2 µg/ml		
catalase	350 ng/ml	350 ng/ml		
glucose	4 mM	4 mM		

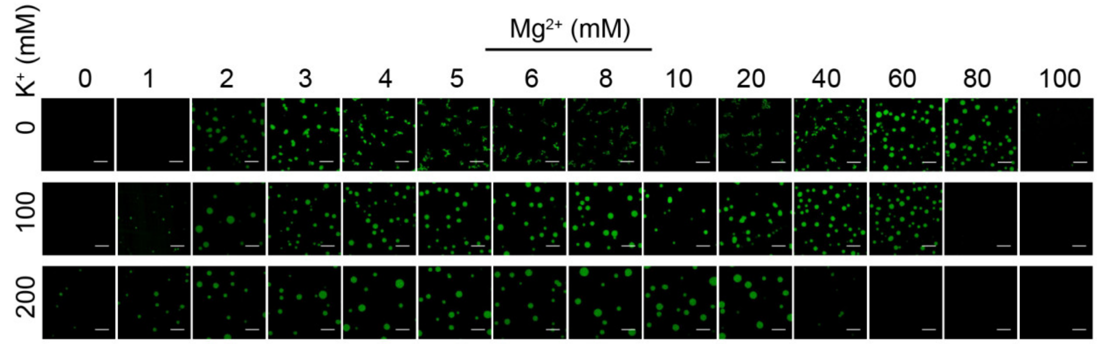

Figure S7. Confocal fluorescence microscopy images of 15-197-601 nucleosome arrays with DNA stained by DyeCycle in different KCl and $MgCl_2$ concentrations. Scale bar represent 10 μm.

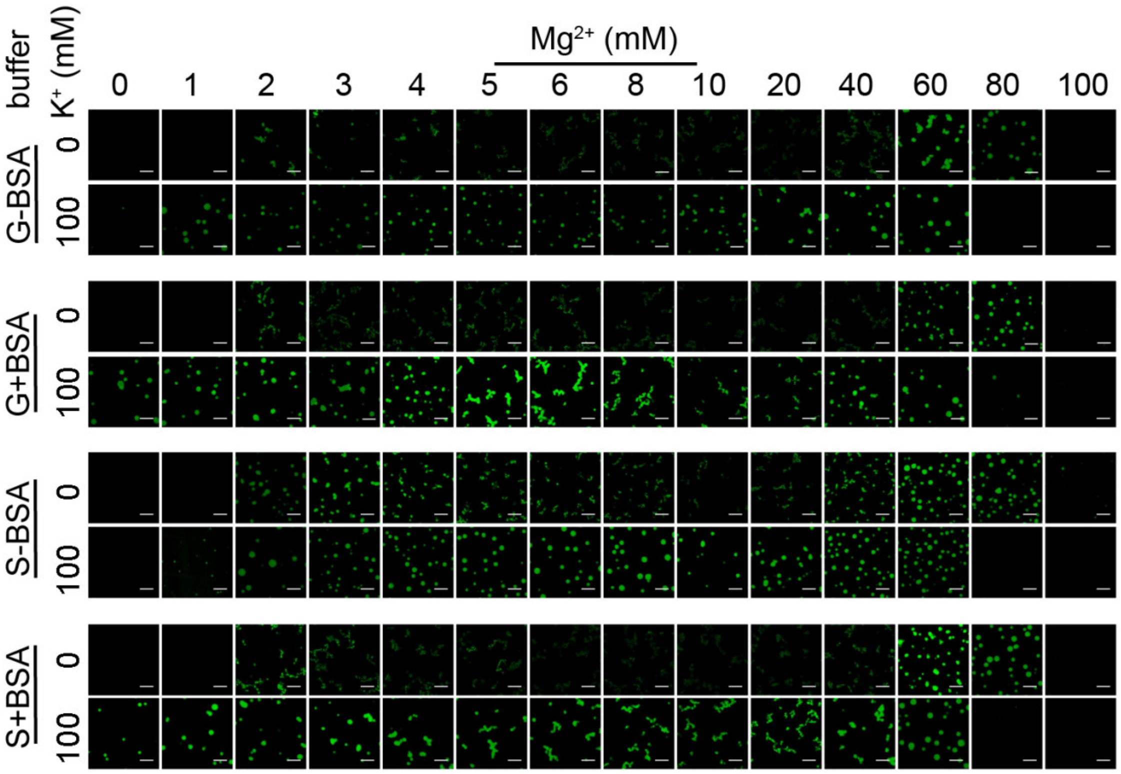

Figure S8. Confocal fluorescence microscopy images of 15-197-601 nucleosome arrays with DNA stained by DyeCycle in the absence and presence of 100 mM KCl at four buffer conditions as a function of $MgCl_2$ concentration. The nucleosome array concentration is 375 nM. The detailed buffer components are listed in Table S1. Scale bar represent 10 μm.

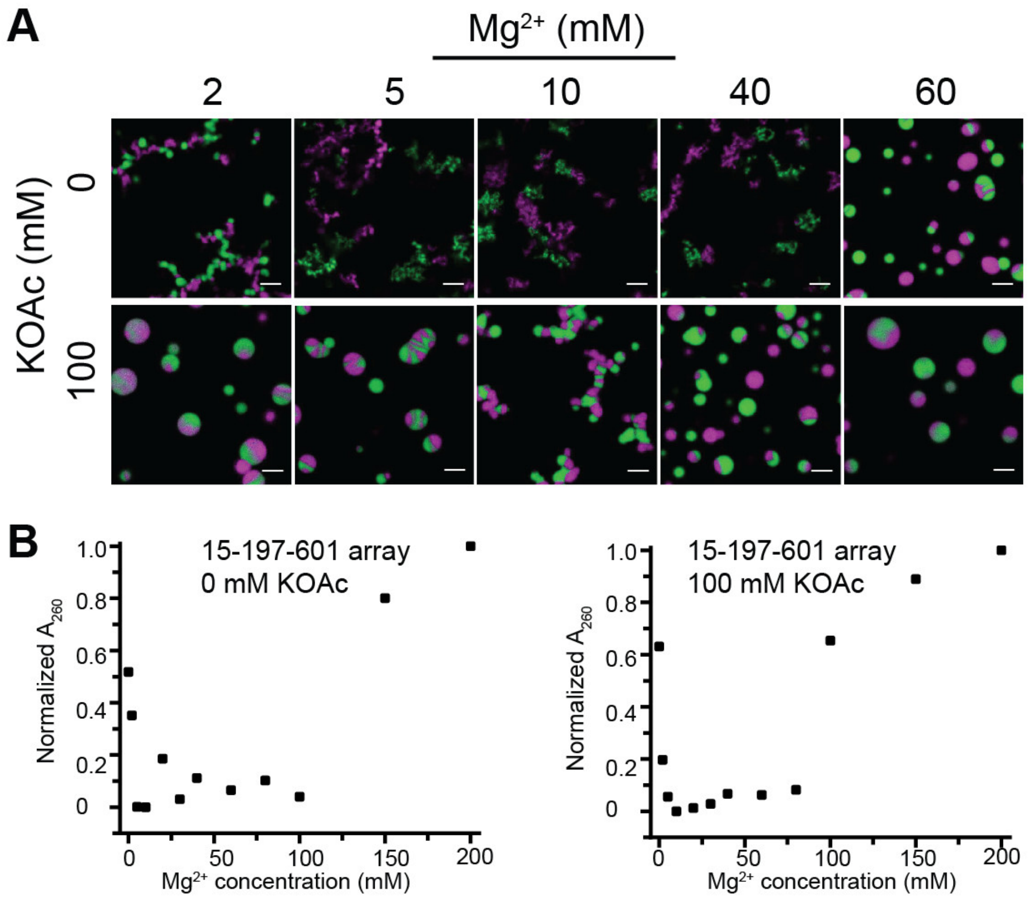

Figure S9. Two-color mixing assay characterization of the solid-like aggregation of 15-197-601 nucleosome arrays in BSA/acetate buffer. (A) Confocal fluorescence microscopy images of samples at conditions with various KOAc and $Mg(OAc)_2$ concentrations are shown. Nucleosomes arrays containing histone H2B T116C labelled with AF488 (green) or AF647 (purple) are treated with phase separation buffer separately and incubated for 30 min before mixing. Then the mixture is incubated for another 20 min before imaging. The array concentration is 375 nM. Scale bar represent 5 μm. (B) Precipitation assay characterization of 15-197-601 nucleosome arrays. The percentage of arrays in the supernatant after centrifugation (10,000 xg for 5 min) are calculated by the normalized absorbance at 260 nm.

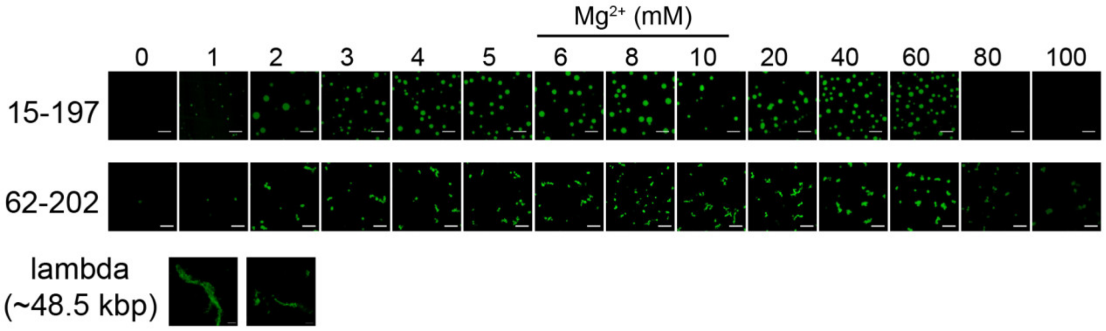

Figure S10. Confocal fluorescence microscopy images of 15-197-601 and 62-202-601 nucleosome arrays were taken for samples in the presence of 100 mM KCl and varisou Mg^{2+} concentrations. Images for lambda DNA arrays were taken for samples at 0 mM (left) and 100 mM (right) KCl and in the absence of Mg^{2+}, where solid-like condensates were observed (same as in the presence of Mg^{2+}). The nucleosome array concentrations are 375 nM. Scale bar represent 10 μm.

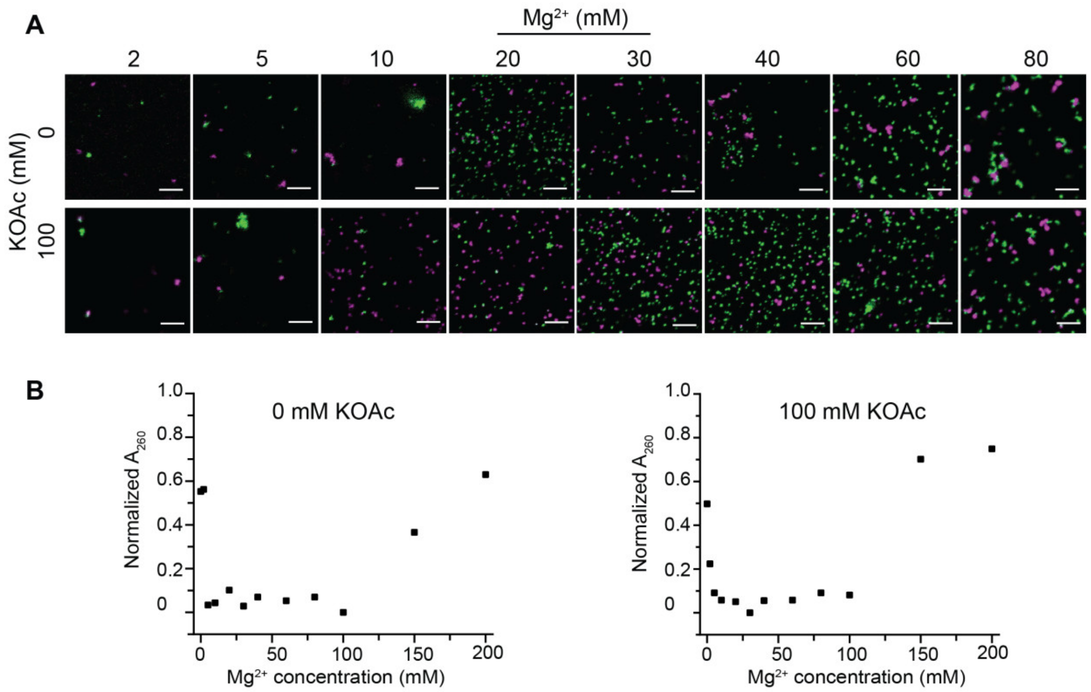

Figure S11. Two-color mixing assay characterization of the solid-like aggregation of 62-202-601 nucleosome arrays in BSA/acetate buffers. (A) Confocal fluorescence microscopy images of samples at conditions with various KOAc and $Mg(OAc)_2$ concentrations are shown. Nucleosomes arrays containing histone H2B T116C labelled with AF488 (green) or AF647 (purple) are treated with phase separation buffer separately and incubated for 30 min before mixing. Then the mixture is incubated for another 20 min before imaging. The array concentration is 375 nM. Scale bar represent 5 μm. (B) Precipitation assay characterization of 62-202-601 nucleosome arrays. The percentage of arrays in the supernatant after centrifugation (10,000 xg for 5 min) are calculated by the normalized absorbance at 260 nm.
