# Supplementary figures and images for: Chromatin Liquid–Liquid Phase Separation (LLPS) Is Regulated by Ionic Conditions and Fiber Length
Source: Cells. 2022 Oct 6;11(19):3145. doi: 10.3390/cells11193145 (PMC9564186; doi:10.3390/cells11193145)

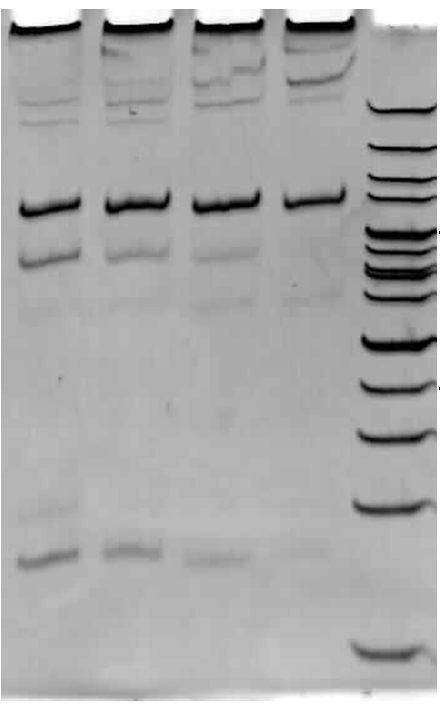

Supplement: Supplementary file 1 [file cells-11-03145-s001.zip › Gels Original Data for submission.png/Figure S1B_Gel_original.png]

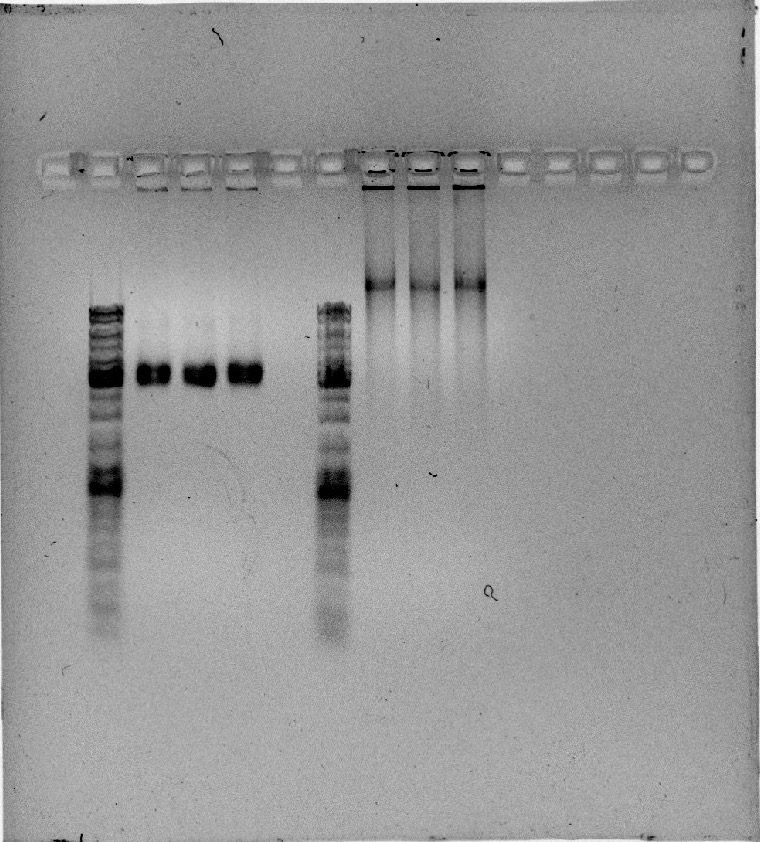

Supplement: Supplementary file 1 [file cells-11-03145-s001.zip › Gels Original Data for submission.png/Figure S2A Gel Original.jpg]

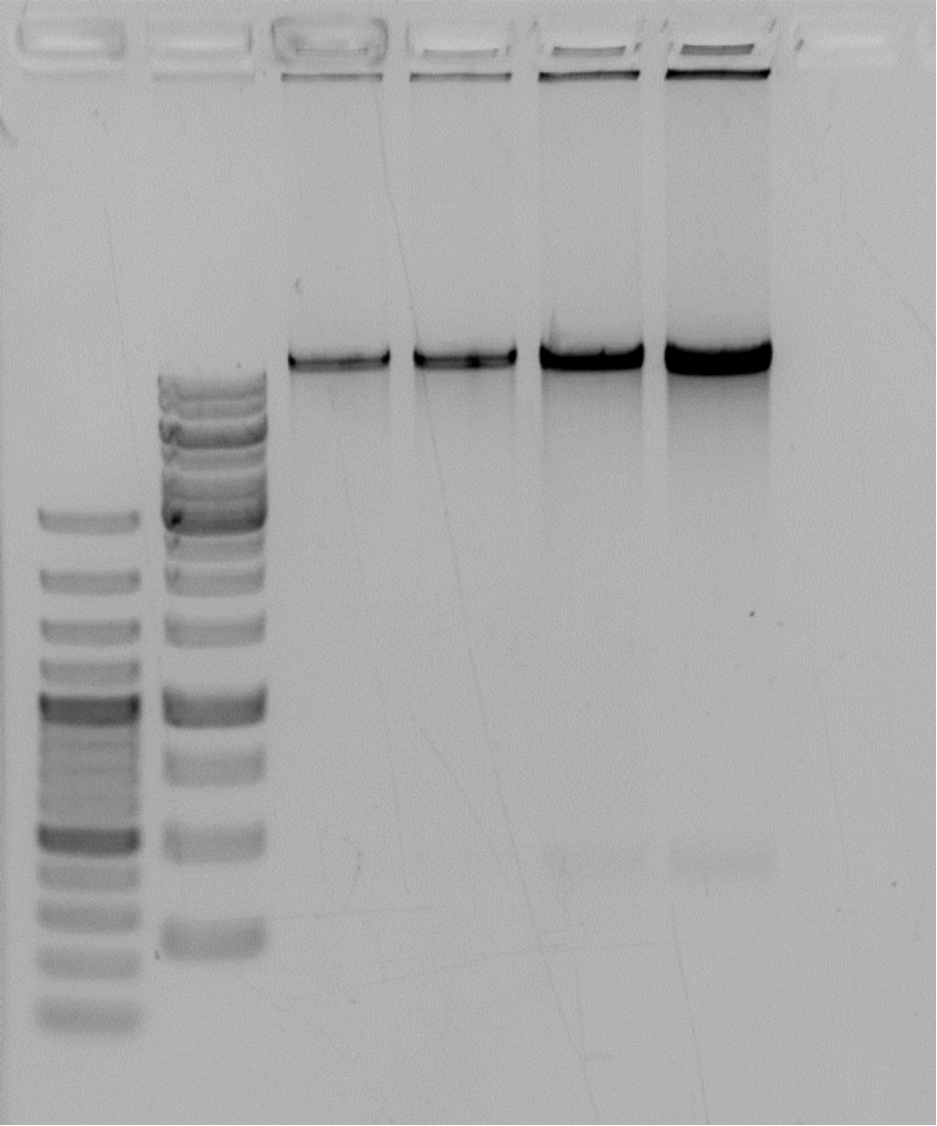

Supplement: Supplementary file 1 [file cells-11-03145-s001.zip › Gels Original Data for submission.png/Figure S2B Original.png]

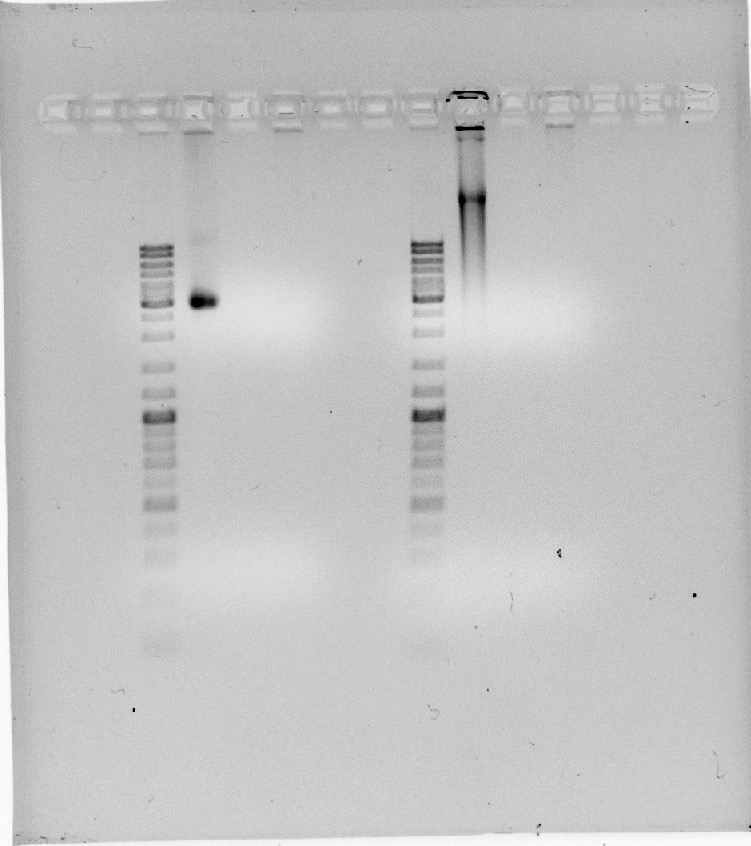

Supplement: Supplementary file 1 [file cells-11-03145-s001.zip › Gels Original Data for submission.png/FIgure S3A Gel Original.jpeg]

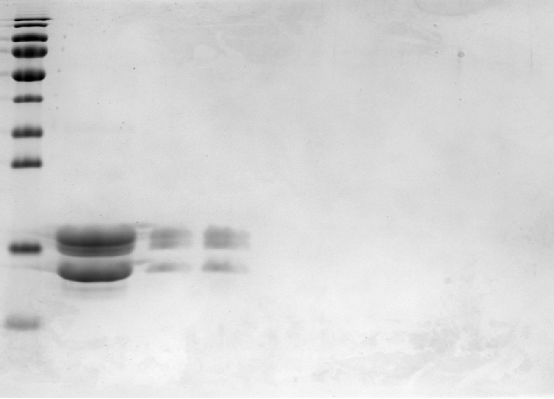

Supplement: Supplementary file 1 [file cells-11-03145-s001.zip › Gels Original Data for submission.png/Figure S3B Gel Original.png]

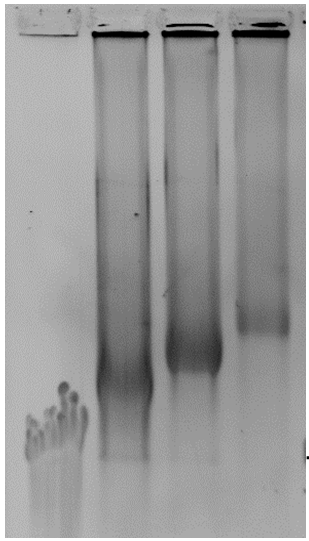

Supplement: Supplementary file 1 [file cells-11-03145-s001.zip › Gels Original Data for submission.png/Figure S4 Gel Original.png]
